# Prevalence and resistance characteristics of multidrug-resistant *Streptococcus pneumoniae* isolated from the respiratory tracts of hospitalized children in Shenzhen, China

**DOI:** 10.3389/fcimb.2023.1332472

**Published:** 2024-01-10

**Authors:** Xing Shi, Sandip Patil, Qing Wang, Zihao Liu, Chunqin Zhu, Heping Wang, Yunshen Chen, Liqiang Li, Liang Yang, Yuejie Zheng, Shaowei Dong, Yanmin Bao

**Affiliations:** ^1^ Department of Respiratory and Critical Care Medicine, Institute of Shenzhen Respiratory Diseases, The First Affiliated Hospital (Shenzhen People’s Hospital), School of Medicine, Southern University of Science and Technology, Shenzhen, China; ^2^ Department of Haematology and Oncology, Shenzhen Children’s Hospital, Shenzhen, Guangdong, China; ^3^ School of Medicine, Southern University of Science and Technology, Shenzhen, Guangdong, China; ^4^ Department of Respiratory Medicine, Shenzhen Children’s Hospital, Shenzhen, Guangdong, China; ^5^ Department of Clinical Microbiology Laboratory, Shenzhen Children’s Hospital, Shenzhen, Guangdong, China; ^6^ Shenzhen Third People's Hospital, National Clinical Research Centre for Infectious Diseases, The Second Affiliated Hospital of Southern University of Science and Technology, Shenzhen, Guangdong, China

**Keywords:** pneumococcus, PCV13, whole genome sequencing, non-vaccine serotype, MDR phenotype

## Abstract

**Background:**

PCV13 introduction in China has led to a significant reduction of vaccine serotype *Streptococcus pneumoniae*. However, non-vaccine serotypes with highly resistance and invasiveness were often reported in the post-pneumococcal conjugate vaccine era and there was regional differences.

**Methods:**

A total of 669 *S. pneumoniae* strains were collected from the respiratory tracts of hospitalized children at Shenzhen Children’s Hospital in 2021 and 2022. Antimicrobial resistance (AMR) characteristics were assessed through antibiotic susceptibility testing performed with the VITEK 2 compact system. AMR genes and single nucleotide polymorphisms (SNPs) in pbp1a, pbp2b, and pbp2x were identified via analysis of whole genome sequencing data. Statistical examination of the data was conducted employing chi-square and Fisher’s exact tests.

**Results:**

We found that non-vaccine serotypes strains had accounted for 46.6% of all the pneumococcal isolated strains. The most common non-vaccine serotype is 23A, with a prevalence rate of 8.9%, followed by 15A (6.6%), 6E (5.7%), 34 (3.2%), and 15B (2.9%). The multidrug resistance rates (MDR) of vaccine serotypes were 19F (99.36%), 19A (100%), 23F (98.08%), 6B (100%), and 6C (100%). Meanwhile, the MDR of non-vaccine serotypes were 15B (100.00%), 6E (100%), 15C (100%), 34 (95.24%), and 23A (98.31%). Resistance rates of 6E to more than six antibiotic classes reached 89.47%, which is similar to 19F (83.33%) and 19A (90%). Unique resistance profiles were also identified for non-vaccine serotypes, including significantly higher resistance to chloramphenicol in 6E, 15B, and 15C than in 19F and 19A. Furthermore, through genome sequencing, we revealed strong correlation of *cat-TC* with chloramphenicol resistance, *patA/patB* with tetracycline resistance, *ermB* and *pmrA* with erythromycin resistance.

**Conclusion:**

The introduction of PCV13 into China from 2017 has led to a shift in the dominant composition of pneumococcal strains. There has been a notable rise and spread of multidrug-resistant non-vaccine serotypes among children. Specifically, the non-vaccine serotype 6E, which was not widely reported in China previously, has emerged. To comprehend the resistance mechanisms, it is crucial to further investigate the molecular and genetic characteristics of these non-vaccine serotypes.

## Introduction

*Streptococcus pneumoniae* (pneumococcus), has a significant threat to children’s health due to their underdeveloped immune systems (CDC, 2022). Pneumococcus frequently colonizes the respiratory tract of children (Fu et al., 2021), leading to various infections, such as community-acquired pneumonia, otitis media, bacteremia, and meningitis (Bogaert et al., 2004). Infections caused by pneumococcus is the leading cause of mortality in children under the age of five globally, responsible for an estimated 294, 000 deaths in HIV-negative children aged 1-59 months in 2015 (Wahl et al., 2018). Over the past two decades, the development of pneumococcal conjugate vaccines (PCVs) has led to a decline in the incidence of invasive pneumococcal disease (IPD) caused by vaccine serotypes (VTs) (Briles et al., 2019; Musher et al., 2022). PCVs also help to reduce antimicrobial resistance (AMR) by preventing infections, reducing antibiotic usage, and promoting herd immunity (Jansen et al., 2021).

However, with the rapid decline of vaccine serotypes after vaccination, there has been a relative increase in the proportion of cases caused by NVT compared to the pre-vaccine era (source: https://www.cdc.gov/pneumococcal/surveillance.html). This highlights the need for increased attention to the epidemiology of NVT serotypes. The prevalence of NVT serotypes varies across different regions, which may be attributed to variations in vaccine coverage, the pre-existing pool of serotypes before vaccination, and the use of different types and dosages of antibiotics. For example, in South Africa, NVTs 15B, 8, and 23B are more commonly associated with pneumococcal disease in children under two years old (Gottenberg et al., 2014). In the United States, NVTs such as 22F, 33F, and 15B/C have become more prevalent (Moore et al., 2016; Balsells et al., 2018). In Asia, NVTs such as 35B and 15A are commonly observed in Japan (Miyazaki et al., 2017), while in Korea, serotypes 10A, 34, and 22F have increased in prevalence after the introduction of PCV13(Kim et al., 2023). In Taiwan, NVTs such as 15A, 23A, 23B, and 34 have been frequently observed (Wu et al., 2020). The emergence of non-vaccine serotypes (NVTs) due to serotype replacement and the development of multi-drug resistance in NVTs pose ongoing challenges (Sings et al., 2019; Wu et al., 2022). Pneumococcus possesses high genome mutation capabilities and the ability to acquire resistance genes from other species by horizontal gene transfer (D’Aeth et al., 2021). Although vaccines offer strong protection against highly pathogenic and highly resistant strains, pneumococcus can still develop vaccine-escape strains that carry multiple resistances within the limits of genetic variation (Azarian et al., 2018). Indiscriminate antibiotic usage has also contributed to the selection pressure for highly resistant bacteria, complicating the treatment of pneumococcal infections. In Asian countries from 2008 to 2009, 59.3% of pneumococcus exhibited multidrug resistance (MDR), with the most prevalent serotypes being 19F, 23F, 19A, 14, and 6B, and the highest MDR rates were found in China at 83.3% (Kim et al., 2012). Importantly, the emergence of MDR in non-vaccine serotypes has caused extensive concern as the proportion of infections increases (Kim et al., 2020; Yamba Yamba et al., 2022). Therefore, it is crucial to monitor and comprehend the impact of PCV13 on serotype replacement and antibiotic resistance in children to inform treatment strategies and guide the development of effective pneumococcal vaccines.

In China, PCV13 was introduced in June 2017, and quickly replaced PCV7 to become the primary pneumococcal conjugate vaccine for children due to its broader serotype coverage and better cost-effectiveness (Ma et al., 2013; Li et al., 2021). Shenzhen is the city with the largest children population and the highest vaccination rate of PCV13 in the south of China. In the clinical data of hospitalized children from 2021 to 2022, we found that the vaccination rate has exceeded 80% in Shenzhen. To promptly and efficiently monitor the characteristics of non-vaccine serotypes in the context of rising vaccination rates, we identified and compared the serotype distribution, resistance profiles, and carried resistance genes of 669 strains collected from the children’s respiratory tract, who were hospitalized because of different kinds of respiratory infection diseases in 2021 and 2022. Our findings revealed a high prevalence of MDR among NVT serotypes, particularly 6E, which exhibited a non-susceptibility rate of 89.47% to more than six classes of antibiotics, akin to 19F. Given the lack of effective defense against NVT strains, monitoring the drug resistance of NVT is of paramount importance. Furthermore, molecular detection demonstrated a strong correlation between non-susceptibility to different antibiotics and the presence of detected genetic materials, indicating a more efficient approach to monitoring changes in resistance trends among prevalent serotypes.

## Materials and methods

This study was conducted in accordance with the guidelines of the Declaration of Helsinki and approved by the Institutional Ethics Committee, Shenzhen Children’s Hospital, reference number: 202200302 which complies with international ethical standards.

### Sputum and BALF culture

A total of 669 non-duplicated (one specimen from one patients) *Streptococcus pneumoniae* strains were isolated from sputum and bronchoalveolar lavage fluid (BALF) samples collected from the children’s respiratory tract in the largest tertiary children’s hospital in south of China, Shenzhen Children’s Hospital, between March, 1, 2021 and March, 31, 2022. The clinical information, including age, sex, source of samples, disease types, vaccination status, etc., is collected.

Sputum and bronchoalveolar lavage fluid (BALF) samples were routinely collected for etiological detection from a diverse patient population with various respiratory illnesses, including acute bronchopneumonia, protracted bacterial bronchitis, bronchiolitis, severe pneumonia, acute bronchitis, chronic pneumonia, bacteremia, and asthma. Upon microscopic examination of the smear, Sputum specimens with white blood cells >25 per low magnification field and squamous epithelial cells <10 per low magnification field are considered satisfactory and subsequently included in the study. The specimens were cultured on blood agar plates, and the resulting colonies were examined for morphology and phenotypic characteristics. The identification of *S. pneumoniae* isolates was performed according to the guidelines provided in the Manual of Clinical Microbiology, 11th edition. Furthermore, the isolates were confirmed using two complementary methods: first, by using automated VITEK 2 system (Biomerieux, France) and second by using mass spectrometry (MALDI-TOF MS, Merier, France). MALDI-TOF MS is a rapid and reliable method for microbial identification that involves the analysis of mass spectra generated from ionised microbial proteins. All strains were preserved in 40% glycerol broth medium at -80°C and were re-culture on 5% horse blood agar under conditions of 5% CO2 and 37°C for 12-15 hours before being subjected to further studies.

### Antibiotic sensitivity testing

The antimicrobial susceptibility test (AST) was performed for all 669 confirmed *S. pneumoniae* isolates using the VITEK 2 compact system (BioMerieux, France) with AST-GP68 card. AST was tested for commonly used antibiotics includes; clindamycin (CLI), cefotaxime (CTX), ceftriaxone (CRO), penicillin (PEN), chloramphenicol (CHL), ertapenem (ETP), meropenem (MEM), erythromycin (ERY), linezolid (LNZ), levofloxacin (LVX), moxifloxacin (MFX), ofloxacin (OFX), tetracycline (TCY), trimethoprim-sulfamethoxazole (SXT), and vancomycin (VAN). The AST results were accurately interpreted according to the Clinical and Laboratory Standards Institute (CLSI) breakpoints 2021 (CLSI, 2021). The results for penicillin were interpreted using non-meningitis oral administration breakpoints, whereby susceptibility was defined as ≤ 0.06 μg/ml, intermediate as 0.12-1 μg/ml, and resistance as ≥2 μg/ml. The classification of multi-drug resistance (MDR) phenotype was performed as described previously by Magiorakos et al., MDR was defined as resistance to at least one agent in three or more antimicrobial groups (Magiorakos et al., 2012). MDR3, MDR4, MDR5, and MDR6 were used to represent non-susceptibility to at least 3, 4, 5, and 6 classes of antimicrobials, respectively.

### Molecular analysis

The whole-genome DNA was extracted using the TianGen Magnetic Bead Soil and Fecal Genomic DNA Extraction Kit from TianGen, China. The extracted DNA was then quantified using the Qubit™ dsDNA BR Assay Kit (Thermo Fisher Scientific). The DNA library was prepared using the TruSeq^®^ DNA PCR-Free Sample Preparation Kit from Illumina, San Diego, CA, USA. Sequencing was performed on an Illumina Novaseq6000 from Illumina, San Diego, CA, USA. To ensure high quality, sequence segments with low quality were removed using Trimmomatic v0.36 (Bolger et al., 2014) with a SLIDINGWINDOW parameter of 4:20 and a MINLEN parameter of 70. The remaining high-quality reads were assembled using SPAdes v3.11 (Prjibelski et al., 2020). For molecular serotyping, raw reads were analyzed using seroBA v1.0.2 (Epping et al., 2018) with the default parameters and a recommended k-mer size of 71. Antimicrobial resistance genes (ARGs) were detected by performing a blast search (Camacho et al., 2009) against the CARD database (https://card.mcmaster.ca). Genes with a nucleotide identity higher than 99% were considered ARGs.

### Statistical analysis

Statistical analysis was performed to compare antibiotic-resistant profiles and genes using the chi-square test and Fisher’s exact test. *p* < 0.05 was considered statistically significant.

## Results

### Epidemiology of pneumococcal infections

**Figure 1:**
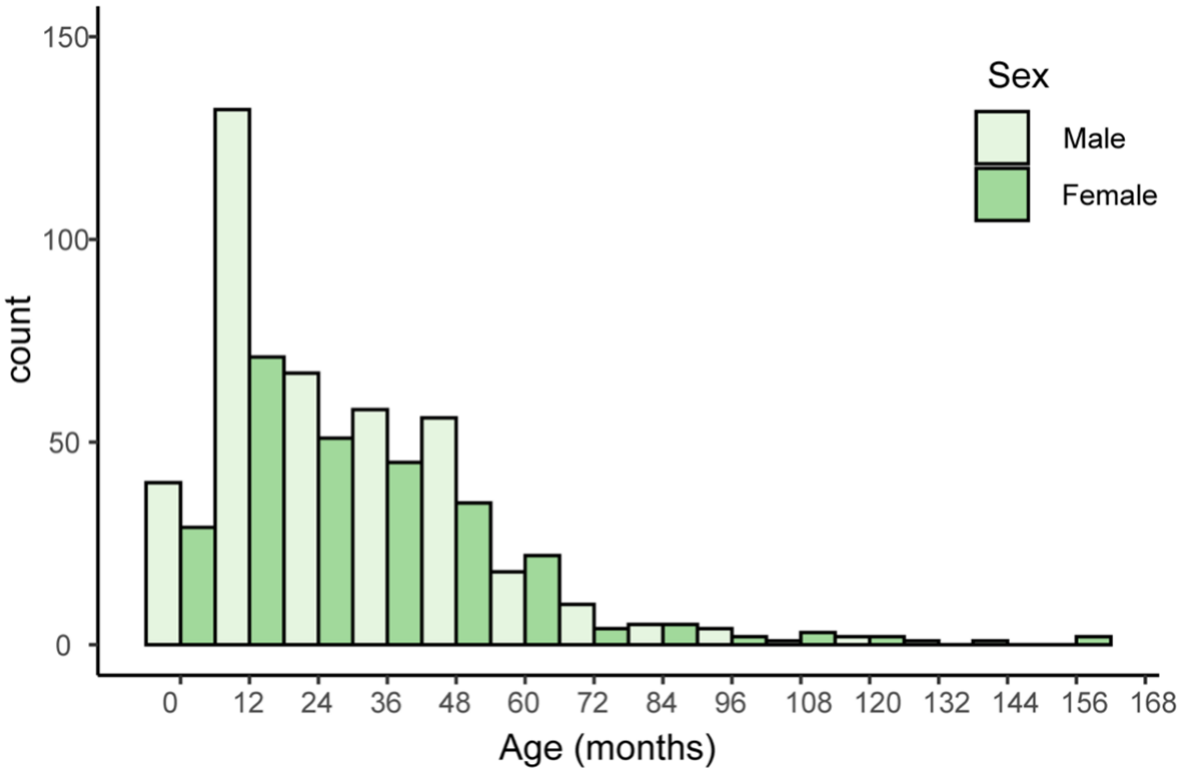
Histogram of patients’ age and sex. The x-axis represents age in months, with 12-month intervals. The y-axis represents the number of individuals with a given serotype within a particular age range.

An overview of demographic information for 669 patients, including age, sex, and sample types provides **Table 1**. The distribution of male (n=396, 59.4%) and female (n = 271, 40.6%) patients is nearly even, with a slight male predominance (**Figure 1**). Sputum samples comprise the majority of samples (n = 613, 91.6%), while bronchoalveolar lavage fluid (BALF) samples account for the remaining 8.4% (n=56). The median age of patients is 24.9 months, with an interquartile range (IQR) of 12.0-43.0 months. All these children were admitted into Shenzhen children’s hospital because of acute or chronic lower respiratory tract infection, including acute bronchopneumonia, protracted bacteria bronchitis and so on.

**Table 1:** Clinical characteristics.

Characteristics	All patients (n=669)
Age, median (IQR), month	24.9 (12.0-43.0)
Sex, n (%)	
Male	396 (59.4)
Female	271 (40.6)
Sources, n (%)	
BALF	56 (8.4)
Sputum	613 (91.6)
Major Diseases	
Acute bronchopneumonia	336
Acute bronchitis	60
Severe pneumonia	36
Bronchiolitis	22
Protracted bacterial bronchitis	21
Acute asthmatic bronchitis	18
Prolonged pneumonia	13
Sepsis	10
Chronic cough	8
Bronchiolitis	8
Others	137

### Distribution of serotypes and coverage of PCVs

**Figure 2:**
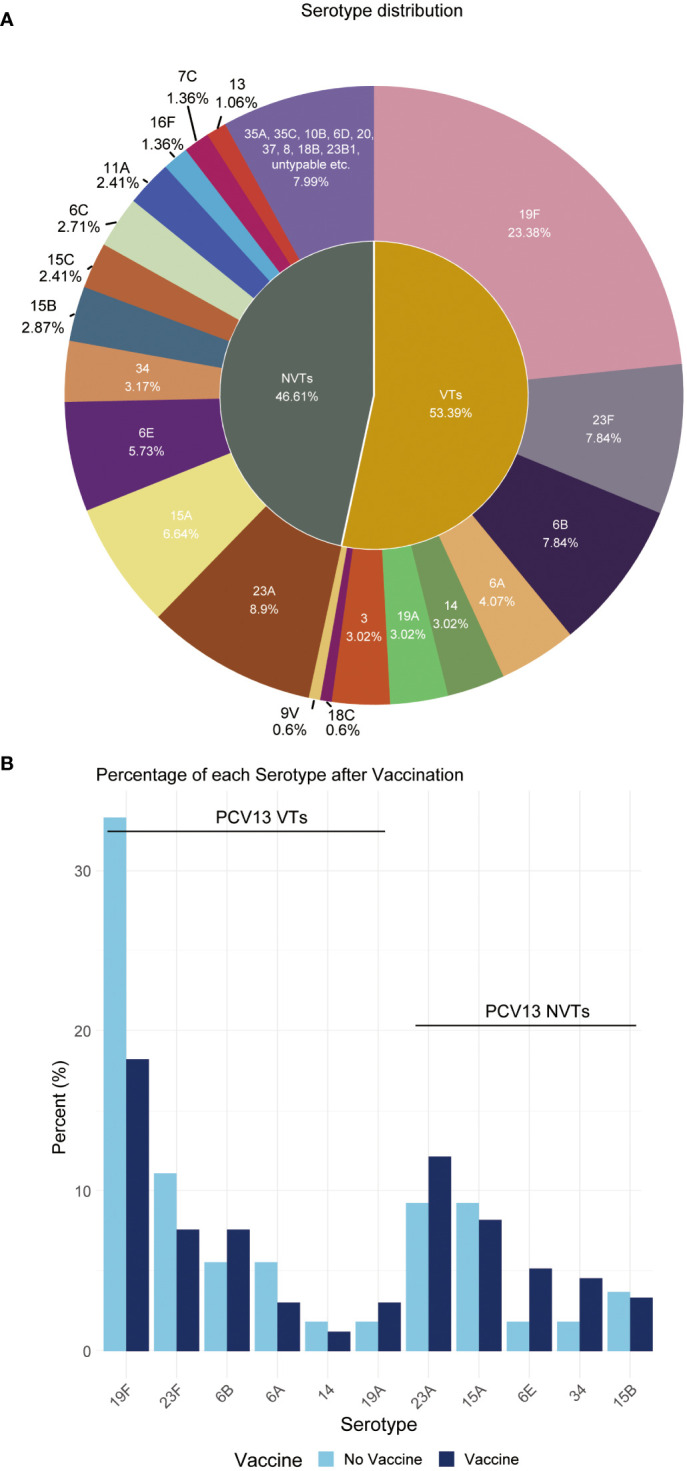
Distribution proportion of *S. pneumoniae* serotypes. **(A)** This pie chart shows the distribution of serotypes observed in all children. Each slice represents a different serotype, with the size of the slice proportional to the percent of that serotype. **(B)** Percentage of each serotype pre- and post-vaccination.

Overall, this study reveals the NVT group has a high detection rate of 46.61% among all 669 samples, which is close to the VT group’s 53.39%. The most prevalent vaccine serotype is 19F, with a prevalence rate of 23.38%, followed by 23F (7.84%), 6B (7.84%), 6A (4.07%), and 14 (3.02%). The most prevalent non-vaccine serotype is 23A, with a prevalence rate of 8.9%, followed by 15A (6.64%), 6E (5.73%), 34 (3.17%), and 15B (2.87%) (**Figure 2A**). The data also indicates a consequential shift in prevalence due to vaccination application, with a decrease in VT strains and a corresponding increase in NVT strains (**Figure 2B**).

### The antimicrobial susceptibility testing

The result of antimicrobial susceptibility analysis of the ten classes of antibiotics was presented in **Table 2**. Based on the AMR profiles, lincosamides and macrolides have the highest rates of non-susceptibility in the major serotypes tested, with rates ranging from 94.4% to 100%, while oxazolidinones, fluoroquinolones, and glycopeptides have extremely low rates of non-susceptibility (**Table 2**). For penicillin, cefotaxime and ceftriaxone, which are often used to treat pneumococcal diseases in China, in common VTs (19F, 19A, 23F, 14, 6A and 6B), the non-susceptibility rate of penicillin were between 95.0% and 97.4%, the non-susceptibility rate of cefotaxime were between 0% and 67.7%, the non-susceptibility rate of ceftriaxone were between 0 and 60.6%, respectively. The serotype of 19F and 19A had the highest drug-resistance to penicillin, cefotaxime and ceftriaxone. In common NVTs (23A, 15A/B/C, 6E and 6C), the non-susceptibility rates of penicillin resistance were between 47.4% and 98.3%, the non-susceptibility rate of cefotaxime were between 0% and 23.5%, the cefotaxime rate of ceftriaxone resistance between 0 and 1.7% respectively. The serotype of 6E and 23A had the highest drug-resistance to penicillin in these common NVTs strain. (**Table 2**). Furthermore, the resistance rate of serotype 6E to chloramphenicol is significantly higher compared to serotype 19F and other serotypes (*p* < 0.001, **Supplementary Table 1**).

**Table 2:** AMR profiles of major serotypes.

	*Antibiotics*	*19F* *(NS %)*	*23F* *(NS %)*	*6B* *(NS %)*	*6A* *(NS %)*	*14* *(NS %)*	*19A* *(NS %)*	*23A* *(NS %)*	*15A* *(NS %)*	*6E* *(NS %)*	*34* *(NS %)*	*15B* *(NS %)*	*15C* *(NS %)*	*6C* *(NS %)*
*Lincosamides*	CLI	152 (100.0)	52 (100.0)	52 (100.0)	27 (100.0)	20 (100.0)	20 (100.0)	59 (100.0)	44 (100.0)	38 (100.0)	21 (100.0)	18 (100.0)	17 (100.0)	18 (100.0)
*Beta-Lactam*	CTX	155 (67.7)	52 (11.5)	52 (3.8)	27 (0.0)	20 (25.0)	20 (35.0)	59 (6.8)	44 (2.3)	38 (2.6)	21 (0.0)	19 (10.5)	17 (23.5)	18 (0.0)
	CRO	155 (60.6)	52 (5.8)	52 (3.8)	27 (0.0)	20 (20.0)	20 (25.0)	59 (1.7)	44 (0.0)	38 (0.0)	21 (0.0)	19 (0.0)	17 (0.0)	18 (0.0)
	PEN	155 (97.4)	52 (96.2)	52 (90.4)	27 (96.3)	20 (95.0)	20 (95.0)	59 (98.3)	44 (72.7)	38 (97.4)	21 (57.1)	19 (47.4)	17 (52.9)	18 (50.0)
*Phenicols*	CHL	155 (0.6)	52 (1.9)	52 (0.0)	27 (0.0)	20 (0.0)	20 (0.0)	59 (0.0)	44 (0.0)	38 (65.8)	21 (0.0)	19 (42.1)	17 (41.2)	18 (5.6)
*Carbapenem*	ETP	155 (1.9)	52 (1.9)	52 (0.0)	27 (0.0)	20 (0.0)	20 (0.0)	59 (0.0)	44 (0.0)	38 (0.0)	21 (0.0)	19 (10.5)	17 (0.0)	18 (0.0)
	MEM	151 (93.4)	51 (66.7)	52 (42.3)	27 (55.6)	20 (95.0)	20 (90.0)	59 (18.6)	44 (40.9)	38 (89.5)	21 (14.3)	19 (36.8)	17 (52.9)	18 (27.8)
*Macrolide*	ERY	152 (99.3)	51 (98.0)	51 (96.1)	25 (100.0)	20 (100.0)	20 (100.0)	55 (98.2)	41 (97.6)	38 (100.0)	19 (100.0)	18 (94.4)	16 (100.0)	17 (100.0)
*Oxazolidinone*	LNZ	155 (0.0)	52 (0.0)	52 (0.0)	27 (0.0)	20 (0.0)	20 (0.0)	59 (0.0)	44 (0.0)	38 (0.0)	21 (0.0)	19 (0.0)	17 (0.0)	18 (0.0)
*Fluoroquinolone*	LVX	155 (0.0)	52 (0.0)	52 (0.0)	27 (0.0)	20 (0.0)	20 (0.0)	59 (0.0)	44 (2.3)	38 (0.0)	21 (0.0)	19 (0.0)	17 (0.0)	18 (0.0)
	MFX	155 (0.0)	52 (0.0)	52 (0.0)	27 (0.0)	20 (0.0)	20 (0.0)	59 (0.0)	44 (0.0)	38 (0.0)	21 (0.0)	19 (0.0)	17 (0.0)	18 (0.0)
	OFX	155 (0.0)	52 (0.0)	52 (0.0)	27 (0.0)	20 (0.0)	20 (0.0)	59 (11.9)	44 (2.3)	38 (0.0)	21 (0.0)	19 (0.0)	17 (0.0)	18 (0.0)
*Tetracycline*	TCY	155 (96.8)	52 (96.2)	52 (96.2)	27 (74.1)	20 (90.0)	20 (100.0)	58 (96.6)	44 (75.0)	38 (89.5)	21 (85.7)	19 (94.7)	17 (94.1)	18 (94.4)
*Folate pathway* *antagonists*	SXT	155 (98.7)	52 (96.2)	52 (100.0)	27 (70.4)	20 (5.0)	20 (100.0)	59 (45.8)	44 (6.8)	38 (92.1)	21 (90.5)	19 (94.7)	17 (94.1)	18 (83.3)
*Glycopeptide*	VAN	155 (0.0)	52 (0.0)	52 (0.0)	27 (0.0)	20 (0.0)	20 (0.0)	59 (0.0)	44 (0.0)	38 (0.0)	21 (0.0)	19 (0.0)	17 (0.0)	18 (0.0)

CLI, clindamycin; CTX, cefotaxime; CRO, ceftriaxone; PEN, penicillin; CHL, chloramphenicol; ETP, ertapenem; MEM, meropenem; ERY, erythromycin; LNZ, linezolid; LVX, levofloxacin; MFX, moxifloxacin; OFX, ofloxacin; TCY, tetracycline; SXT, trimethoprim-sulfamethoxazole; VAN, vancomycin.

### MDR profiling

The MDR combination patterns vary across different serotypes, as demonstrated in **Table 3**. The combination of CLI|ERY|TCY is the most frequently observed MDR pattern across all serotypes. The specific MDR combinations differ among serotypes due to resistance to other specific antibiotics. For instance, within the 19F serotype, the most common MDR combination is CLI|ERY|TCY|PEN|SXT|MEM|CRO|CTX (83/156), while within the 6E serotype, the most common MDR combination is CLI|ERY|TCY|PEN|SXT|MEM|CHL (20/38). Through comparison of this combination, we found that chloramphenicol replacing ceftriaxone and cefotaxime constitutes the most common MDR combination of 6E.

**Table 3:** The common antimicrobial resistance patterns of pneumococcus.

	*Total*	*19F*	*23F*	*6B*	*6A*	*14*	*19A*	*23A*	*15A*	*6E*	*34*	*15B*	*15C*	*6C*
*CLI/ERY/TCY*	10								6	1		1	1	1
*CLI/ERY/TCY/SXT*	23	2	1	5	1		1							7
*CLI|PEN|ERY*	11				7			1	2					1
*CLI|PEN|ERY|TCY*	38	1	1					27	6	2				1
*CLI|PEN|ERY|TCY|SXT*	65	6	13	21	2		1	6	3	1	8	2		2
*CLI|PEN|ERY|TCY|SXT|MEM*	134	34	26	21	14		1	7		9	1	3	4	4
*CLI|PEN|ERY|TCY|MEM*	32	1			1	12			16		2			
*CLI|PEN|ERY|TCY|SXT|MEM|CRO|CTX*	91	83	1	1			5	1						
*CLI|PEN|ERY|TCY|SXT|MEM|CTX*	23	10	3				2	3				1	4	
*CLI|PEN|ERY|TCY|SXT|MEM|CHL*	21									20				1

CLI, clindamycin; CTX, cefotaxime; CRO, ceftriaxone; PEN, penicillin; CHL, chloramphenicol; ETP, ertapenem; MEM, meropenem; ERY, erythromycin; LNZ, linezolid; LVX, levofloxacin; MFX, moxifloxacin; OFX, ofloxacin; TCY, tetracycline; SXT, trimethoprim-sulfamethoxazole; VAN, vancomycin.

### MDR patterns of NVT serotype 6E compared to VT serotype 6B

As an NVT serotype, 6E has a similar occurrence rate compared to the VT serotype 6B, as shown in **Figure 2A** However, 6E exhibits significantly higher rates of multidrug resistance (MDR) than 6B, particularly in MDR5, and MDR6, with rates of 92.1%, and 89.5%, respectively, compared to 6B’s rates of 84.6%, and 42.3%, respectively (**Table 4**). Additionally, 6E shows a higher resistance rate to antibiotics such as chloramphenicol and meropenem, where 25/38 and 34/38 bacterial strains are non-susceptible, respectively, compared to 6B with 0/52 and 22/52 non-susceptible strains (*p* < 0.01, **Table 5**). These findings suggest that both distinct MDR patterns and vaccine use could play crucial roles in shaping the future prevalence of serotypes 6E and 6B. Therefore, further analysis and monitoring of the spreading of these serotypes are necessary to fully understand their eventual impact.

**Table 4:** MDR rate of major serotypes.

*Serotypes*	*Total strains*	*No. of MDR3 strains*	*No. of MDR4 strains*	*No. of MDR5 strains*	*No. of MDR6 strains*	*MDR3%*	*MDR4%*	*MDR5%*	*MDR6%*
*6E*	38	38	37	35	34	100.00	97.37	92.11	89.47
*19F*	156	155	154	151	130	99.36	98.72	96.79	83.33
*19A*	20	20	20	19	18	100.00	100.00	95.00	90.00
*23F*	52	51	51	48	33	98.08	98.08	92.31	63.46
*15C*	17	17	16	15	8	100.00	94.12	88.24	47.06
*6B*	52	52	50	44	22	100.00	96.15	84.62	42.31
*15B*	19	19	17	15	6	100.00	89.47	78.95	31.58
*6A*	27	27	20	17	14	100.00	74.07	62.96	51.85
*14*	20	19	19	19	0	95.00	95.00	95.00	0.00
*23A*	59	58	54	24	18	98.31	91.53	40.68	30.51
*6C*	18	18	15	7	5	100.00	83.33	38.89	27.78
*34*	21	20	18	11	1	95.24	85.71	52.38	4.76
*15A*	44	37	27	20	0	84.09	61.36	45.45	0.00

CLI, clindamycin; CTX, cefotaxime; CRO, ceftriaxone; PEN, penicillin; CHL, chloramphenicol; ETP, ertapenem; MEM, meropenem; ERY, erythromycin; LNZ, linezolid; LVX, levofloxacin; MFX, moxifloxacin; OFX, ofloxacin; TCY, tetracycline; SXT, trimethoprim-sulfamethoxazole; VAN, vancomycin.

**Table 5:** Comparison of antibiotic resistance of 6E and 6B.

	6E			6B			p
	**Susceptible**	**Intermediate**	**Resistant**	**Susceptible**	**Intermediate**	**Resistant**
**CHL**	13	0	25	52	0	0	< 0.001
**CLI**	0	0	38	0	0	52	1.000
**CRO**	38	0	0	50	2	0	0.507
**CTX**	37	1	0	50	1	1	1.000
**ERY**	0	0	38	2	0	49	0.505
**ETP**	38	0	0	52	0	0	1.000
**LNZ**	38	0	0	52	0	0	1.000
**LVX**	38	0	0	52	0	0	1.000
**MEM**	4	31	3	30	21	1	< 0.001
**MFX**	38	0	0	52	0	0	1.000
**OFX**	38	0	0	52	0	0	1.000
**PEN**	1	32	5	5	44	3	0.245
**SXT**	3	0	35	0	42	10	< 0.001
**TCY**	4	1	33	2	1	49	0.422
**VAN**	38	0	0	52	0	0	1.000

CLI, clindamycin; CTX, cefotaxime; CRO, ceftriaxone; PEN, penicillin; CHL, chloramphenicol; ETP, ertapenem; MEM, meropenem; ERY, erythromycin; LNZ, linezolid; LVX, levofloxacin; MFX, moxifloxacin; OFX, ofloxacin; TCY, tetracycline; SXT, trimethoprim-sulfamethoxazole; VAN, vancomycin.

### Characteristics of antibiotic resistance genes

**Figure 3:**
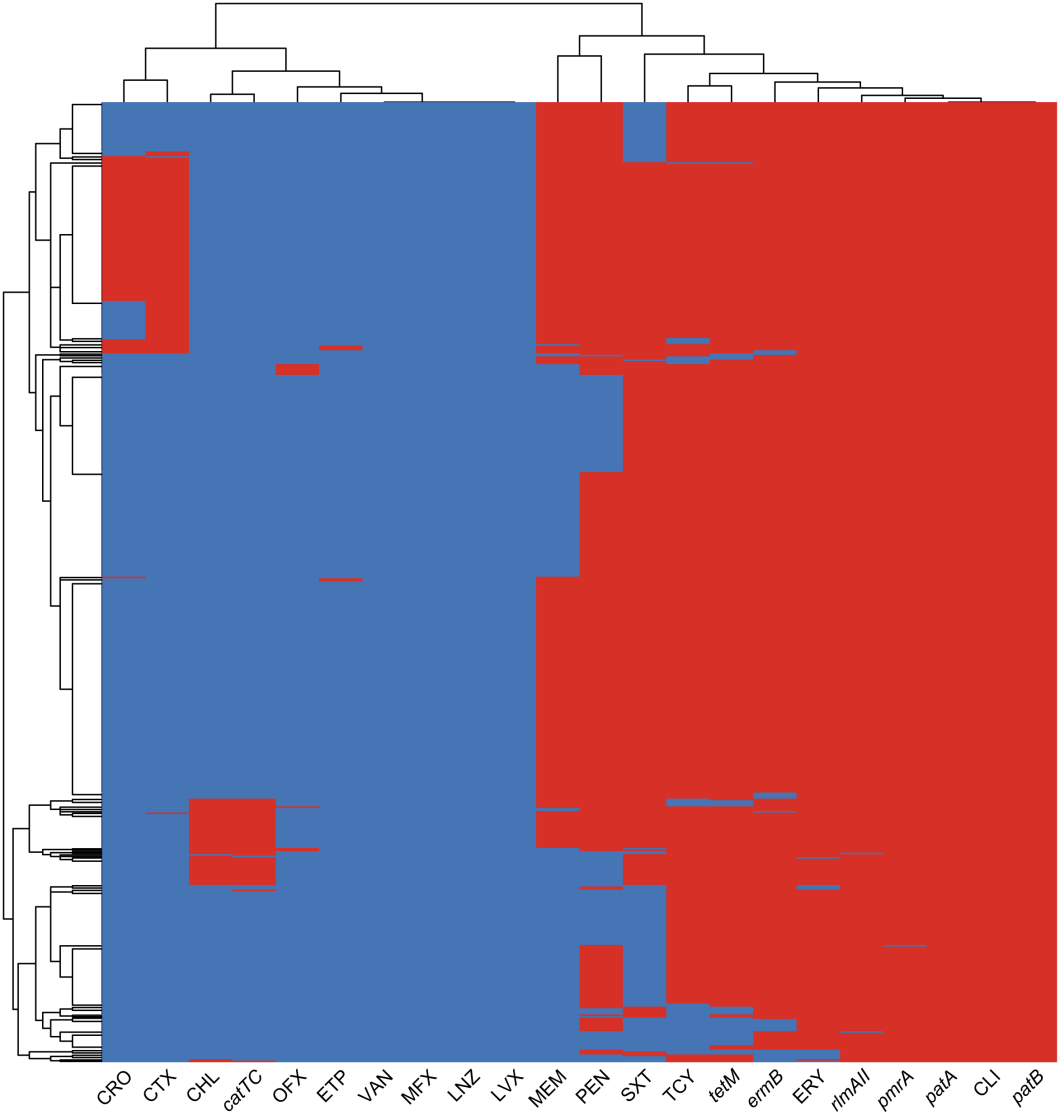
The heatmap of antimicrobial resistance phenotypes and resistance genes co-detection. The heatmap displays the clustering results of ARGs and AMR profiles in bacterial isolates. The color of each cell in the heatmap indicates the non-susceptibility (red), and susceptibility (blue) of a particular kind of antibiotic, and the presence (red) or absence (blue) of a particular ARG in a *S. pneumoniae* isolate.

In our study, we observed a strong correlation between the detection of antibiotic resistance genes and the corresponding antibiotic resistance profiles, as illustrated in **Figure 3**. Specifically, the *cat-TC* gene exhibited the highest consistency of 99.4% with chloramphenicol non-susceptibility. This was followed by the *tetM* gene which showed consistency of 97.3% with tetracycline non-susceptibility. And *ErmB* gene showed consistency of 96.5% with erythromycin non-susceptibility, while the *PatA/PatB* gene showed a consistency of 92.3% with tetracycline non-susceptibility. Lastly, the *pmrA* gene exhibited a correlation of 89.1% with erythromycin non-susceptibility. These findings suggest that the identification of specific antibiotic resistance genes can serve as reliable predictors of the corresponding antibiotic resistance profiles. In addition, we found dozens important polymorphic loci of PBP (Penicillin-binding protein) genes that are strongly associated with penicillin susceptibility, prominently including pbp2b’s A1336G (Thr446Ala), T1542C, pbp1a’s T1740A and pbp2x’s T/G1713A (**Supplementary Figure 1**; **Supplementary Table 2**). These polymorphic sites can be used to accurately predict penicillin susceptibility.

## Discussion

In Chinese mainland, PCV13 was introduced in 2017. However, it has not been incorporated into the national immunization schedule, resulting in a low nationwide vaccination rate. Shenzhen, the largest city in southern China and a significant economic hub, presents a contrasting picture with an estimated 50% of children receiving the PCV13 vaccine, according to data from the Chinese CDC. However, in the clinical data we gathered from Shenzhen Children’s Hospital for the years 2021 to 2022, pertaining to hospitalized children, it was observed that the vaccination rate among these patients has surpassed 80%. Given this, it becomes crucial to investigate the potential changes in the prevalence of NVTs in Shenzhen in the post-PCV13 era.

As the prevalence of NVT serotypes increase, we also found a concurrent shift in the overall drug resistance profile. A study in Taiwan found that the non-susceptibility of pneumococcus to penicillin, cefotaxime and ceftriaxone was decreased along with the decrease of serotypes 19F and 19A (Huang et al., 2022), which is consistent with our data that 19F and 19A provide the majority non-susceptibility to penicillin, cefotaxime and ceftriaxone, while the major NVT serotypes are much more susceptible to these three kinds of antibiotics. It is worth noting that NVT resistance possesses distinctive uniqueness which leads to the emergence of multidrug-resistant serotypes (Lo et al., 2022). Our data reveals that in all airway samples, 65.8% of strains of serotype 6E, 42.1% of strains of serotype 15B, and 41.2% of strains of serotype 15C are non-susceptible to chloramphenicol, while most VT serotypes are susceptible to this antibiotic. Serotype 6E, the third most prevalent NVT serotype, exhibits a comparable level of MDR to serotypes 19F and 19A, with a 89.5% non-susceptibility rate to six classes of antibiotics. With the increasing prevalence of serotypes 6E and 15B/C detected in IPD, it is crucial to closely monitor their MDR profiles (Lo et al., 2022).

The genetic flexibility of Pneumococci allows for rapid adaptation to changing environmental pressures, including vaccine selection pressure (Nj et al., 2013). Resistance to specific antibiotics can result from mutations and genetic recombination in chromosomal genes, such as *pbp2x, pbp2b, and pbp1a*, which confer resistance to beta-lactam antibiotics (Mosadegh et al., 2022). Additionally, antibiotic resistance genes are often carried on mobile genetic elements (MGEs). For example, Tn916-type integrative and conjugative elements (ICEs) can transfer resistance genes for tetracycline and erythromycin, such as *tetM* and *ermB* (Cochetti et al., 2007; Croucher et al., 2009). The *cat-TC* gene, which confers resistance to chloramphenicol, is often carried on the transposon Tn5253, which serves as an important hotspot for insertion sites (Shoemaker et al., 1979; Ayoubi et al., 1991; Santoro et al., 2019). In our data, we found that the resistant genes *pmrA*, *ermB*, *rlmA* ^II^, *pafA*/*B*, and *tetM* are commonly present in streptococcus, which contribute to the widespread multidrug resistance patterns against macrolides, tetracyclines, and clindamycin. In addition, our study highlights the significance of both synonymous and non-synonymous mutation sites in PBP alleles. The non-synonymous mutation, specifically the PBP2b Thr446Ala, which is known to contribute to adaptive resistance to penicillin (Pagliero et al., 2004), holds the highest predictive importance value. However, the results also suggest that synonymous mutation sites could be valuable predictors of penicillin resistance, potentially due to their impact on PBP proteins’ expression or function. In summary, to effectively control the spread of antibiotic resistance in *S. pneumoniae*, a multifaceted approach is required. This includes the judicious use of antibiotics, monitoring resistance patterns, and developing new strategies for prevention and treatment of infections caused by resistant strains. The combination of antibiotic resistance genes and chromosomal mutations in different serotypes can result in varying resistance profiles, highlighting the need for continued monitoring of antibiotic resistance in pneumococcal populations. Additionally, due to the limited coverage of vaccines, it is essential to pay attention to multidrug resistance in NVT serotypes of *S. pneumoniae*, as they lack specific preventive measures. Thus, it is necessary to strengthen monitoring and treatment of NVT serotypes of *S. pneumoniae* to reduce their threat to public health, particularly serotype 6E, which exhibits a high level of MDR and is not currently covered by any existing vaccine.

**Limitation:** It is important to note that this was a retrospective, single-center study and the isolated strains were collected within a year from March 2021 to March 2022. The provided data cannot represent the overall situation of pneumococcal vaccine usage and serotype prevalence in China. However, given the increasing vaccination rates year by year, it can serve as a basis for predicting future trends. Direct comparison data on the drug resistance of pre-vaccine serotypes were not available, and our findings only provide the antimicrobial drug resistance rate for airway colonized serotypes, without inclusion of invasive strains. To address these limitations, we plan to extend our work by including samples from other parts of the country and invasive strains to gain a more comprehensive understanding of the drug resistance patterns. This could potentially provide valuable insights into the emergence of multidrug-resistant non-vaccine type serotypes and guide the development of effective prevention and treatment strategies.

## Data availability statement

The original contributions presented in the study are included in the article/**Supplementary Material**. Further inquiries can be directed to the corresponding authors.

## Ethics statement

The studies involving humans were approved by Shenzhen Children’s Hospital Institutional Animal Care and Use Committee. The studies were conducted in accordance with the local legislation and institutional requirements. Written informed consent for participation in this study was provided by the participants’ legal guardians/next of kin.

## Author contributions

XS: Conceptualization, Data curation, Validation, Writing – original draft. SP: Project administration, Writing – review & editing. QW: Data curation, Validation, Writing – original draft. ZL: Formal Analysis, Methodology, Validation, Writing – review & editing. CZ: Formal Analysis, Validation, Writing – review & editing. HW: Methodology, Writing – review & editing. YC: Validation, Writing – review & editing. LL: Data curation, Project administration, Writing – review & editing. LY: Project administration, Writing – review & editing. YZ: Validation, Writing – review & editing. SD: Conceptualization, Methodology, Writing – review & editing. YB: Conceptualization, Funding acquisition, Project administration, Writing – original draft.

## Supplementary material

Supplementary Table 1 Comparison of antibiotic resistance of 6E and 19F.

Supplementary Table 2 Polymorphic loci of pbp genes associated with penicillin resistance.

Supplementary Figure 1 Penicillin-related pbp gene polymorphic sites and their odd ratios.
