# Supplementary figures and images for: Prevalence and resistance characteristics of multidrug-resistant Streptococcus pneumoniae isolated from the respiratory tracts of hospitalized children in Shenzhen, China
Source: Front Cell Infect Microbiol. 2024 Jan 10;13:1332472. doi: 10.3389/fcimb.2023.1332472 (PMC10806184; doi:10.3389/fcimb.2023.1332472)

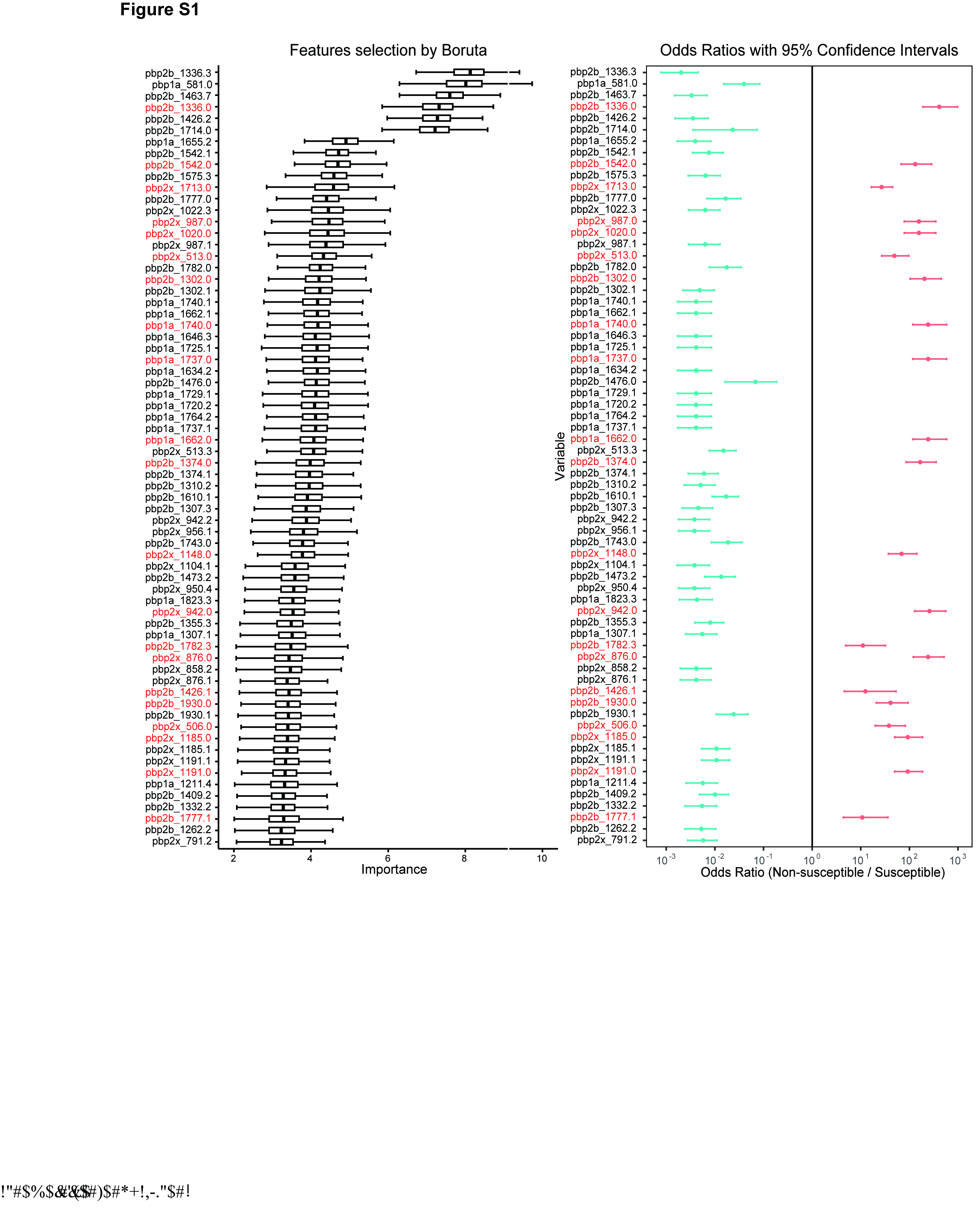

Supplement: Supplementary Figure 1 — Penicillin-related pbp gene polymorphic sites and their odd ratios. [file Image_1.tif]
